# Long-term survivorship and results in lower limb arthroplasty: a registry-based comparison study

**DOI:** 10.1186/s12891-023-06398-7

**Published:** 2023-04-19

**Authors:** Kevin Ki-Wai Ho, Wai-Wang Chau, Lawrence Chun-Man Lau, Jonathan Patrick Ng, Kwok-Hing Chiu, Michael Tim-Yun Ong

**Affiliations:** 1 Department of Orthopaedics and Traumatology, Chinese University of Hong Kong, Prince of Wales Hospital, Shatin, Hong Kong SAR, China; 2 Department of Orthopaedics and Traumatology, Prince of Wales Hospital, Hong Kong SAR, China

**Keywords:** Joint Replacement, Arthroplasty, Registries, Knee, Hip, Patient reported outcome measures

## Abstract

**Introduction:**

Popularity of joint replacement surgery due to ever aging population surges the demand for a proper national joint registry. Our Chinese University of Hong Kong – Prince of Wales Hospital (CUHK-PWH) joint registry has passed the 30^th^ year. The aims of this study are 1) summarize our territory-wide joint registry which has passed the 30th year since establishment and 2) compare our statistics with other major joint registries.

**Methods:**

Part 1 was to review the CUHK-PWH registry. Demographic characteristics of our patients who underwent knee and hip replacements had been summarized. Part 2 was a series of comparisons with registries from Sweden, UK, Australia and New Zealand.

**Results:**

CUHK-PWH registry captured 2889 primary total knee replacements (TKR) (110 (3.81%) revision) and 879 primary total hip replacements (THR) (107 (12.17%) revision). Median Surgery time of TKR was shorter than THR. Clinical outcome scores were much improved after surgery in both. Uncemented of hybrid in TKR were most popular in Australia (33.4%) and 40% in Sweden and UK. More than half of TKR and THR patients showed the highest percentage with ASA grade 2. New Zealand reflected the best cumulative percentage survival 20 years after surgery of 92.2%, 76.0%, 84.2% survivorship 20 years after TKR, unicompartmental knee replacement (UKR) and Hip.

**Conclusion:**

A worldwide accepted patient-reported outcome measure (PROM) is recommended to develop to make comparisons among registries and studies feasible. Completeness of registry data is important and useful to improve surgical performance through data comparisons from different regions. Funding from government on sustaining registries is reflected. Registries from Asian countries have yet to be grown and reported.

**Supplementary Information:**

The online version contains supplementary material available at 10.1186/s12891-023-06398-7.

## Introduction

World Health Organization (WHO) released a fact sheet titled “Ageing and health” on 1 October 2022 projecting that 1 in 6 people in the world will be aged 60 years or over by year 2030, and the proportion of the world's population over 60 years will nearly double from 12 to 22% [1]. The advancing age accompanying with the ageing trend means the added living years are dominated by declined physical capacity [1]. In an earlier WHO report titled “Universal Health Coverage and Ageing”, the demand for restorative surgeries (knee/hip replacement) will keep increasing which needs to redesign the age-friendly benefits packages to include the related types of surgeries and subsequent interventions [2]. Together with the popularity of osteoarthritis occurrence in elderly due to the ageing population around the world [3–6], a national registry on joint replacement (arthroplasty) aims at 1) recording, monitoring, analyzing and reporting on surgical and patient outcome performances, 2) collecting a life-long profile of patients’ data from registry entry, primary surgery, revision if any, survivorship, and patient health-related outcomes, 3) providing national statistics on joint replacement as the registry grows which can eventually provide national statistics for reviewing and policy reviewing and making, and 4) comparing the national registry data with registries around the world by common information [7–10].

The world’s first knee replacement registry was initiated in 1975 in Sweden being a product following a prospective nationwide knee arthroplasty pilot study [11]. The first hip replacement registry was also initiated by the Swedish Orthopaedic Association in 1979, although the multi-centre study initiated by Ahnfelt and colleagues used the data collected since 1967 [12]. This report was one of its kind detailing the 4664 first hip revisions performed in Sweden from 1979 through 1986. Since then, similar joint registries have been rapidly proliferating around the world. In Western countries, the trend was started in Norway (also known as Norwegian Joint Registry, established in 1987) [13], US (community-based registry in 1991 and national registry in 1995) [14], Denmark (1997) [15], New Zealand (established in 1998 and nationalized in 1999) [16], Canada (2000) [17], England and Wales (2003) [18]. Joint registries were also happening in Asian, Middle East and African countries and regions, such as Japan (2002) [19], Malawi (2005) [20], Egypt (2007) [21, 22], Pakistan (2014) [14, 23], Iran (2014) [24], India (2005) [25], Taiwan (2016) [26], and Thailand in association with ASEAN Arthroplasty Association (AAA) (2019) [27].

A joint registry is said to be useful and successful to advance joint replacement surgical technique and strategy without putting tremendous efforts to maintain the data quality and integrity over the years. The data can assist decision-makers, academia and industry professionals [28]. In Hong Kong, our territory-wide joint registry was established in line with the establishment of our adult joint replacement centre since 1985. The purpose of setting up the joint registry is to record patient information and provide data on the performance and longevity of replacement joint implants and surgery outcomes. Striving to maintain a good joint registry aims at determining the incidence of primary, revisions, or re-operations; surgical details; types and number of revisions, if any; reasons for revision; types of patients (usually knee osteoarthritis (OA) or rheumatoid arthritis (RA); type of implant; and survival rate of implants. Further calculations on survival functions for different demographic characteristics e.g., age at operation, sex, body height and body weight, drinker and so forth. Similar to major joint registries, our registry also collects patient reported outcome measures (PROMs). PROMs have been developing very fast because patients increased their awareness of their quality of life after joint replacements. PROMs specific for knee arthroscopy (Knee Society Knee Score (KKS) and Knee Society Function Score (KFS), and hip arthroscopy (Harris Hip Score (HHS) are routinely administered in our centre.

Our joint registry has passed the 30^th^ years, therefore, this study aims to review our data collection, surgical performance, and patients’ outcomes and longevity. The second objective was to compare our statistics with other major joint registries through commonly collected items.

## Materials and Methods

The flow of this study was divided into 2 parts. Part 1 was the review on our Chinese University of Hong Kong—Prince of Wales Hospital Joint Registry (CUHK-PWH Joint Registry or CUHK-PWH Registry or CUHK-PWH in short) since September 1985. Part 2 was a series of comparisons among different major joint registries, namely The Swedish Arthroplasty Register from Sweden (Sweden), National Joint Registry from the UK (UK), Australian Orthopaedic Association National Joint Replacement Registry (Australia or Aus), and The New Zealand Joint Registry. Registries from Sweden (NZ), UK, Australia and New Zealand publish their reports annually or biannually. In this study, we chose the registry reports which analysed data for the period until December 2020, regardless of the year of publication. Therefore, we picked the 2021 annual reports from Sweden, UK and Australia because these reports analysed data up to December 2020 and picked the 2022 annual report from New Zealand (published in December 2021) because this report analysed data up to December 2020.

### Part 1: Review of the CUHK-PWH joint registry

#### Data structure

The dataset structure of CUHK-PWH Joint Registry was divided into 2 parts: 1) data variables common for both Knee and Hip and 2) data variables collected from knee replacement or hip replacement (i.e., specific variables).

#### Common variables

These are non-specific variables. These are demographical characteristics, surgical details and last seen status. Typical examples of variables were personal identification number, sex, age, past medical history, date and duration of surgery, American Society of Anesthesiologists (ASA) Classification, duration of hospital stay, any revision and reason(s) of revision, last seen status (Alive/Death), date and duration of latest follow-up. Details were found in Appendix 1.

#### Knee and hip replacement specific variables

Those variables refer to “joint specific”. Most of the variables are surgery related (obviously the surgical procedures were completely different from each other). Apart from previous knee or hip operation, implant used, implant dimensions, and patient reported outcome measures (Knee Society Score and Knee Society Function Score for Knee replacement surgery and Harris Hip Score for Hip replacement surgery collected before and after surgery) were collected in the respective datasets. Details of the variables were listed in Appendix 2 and 3 respectively.

### Part 2: Comparing with major registries

Being mentioned, 4 internationally recognized registries were introduced and compared with the CUHK-PWH Joint Registry, namely Swedish Arthroplasty Register – Annual Report 2021, National Joint Registry from the UK – 18^th^ Annual Report Annual Report (2021), Australian Orthopaedic Association National Joint Replacement Registry – Annual Report 2021, and The New Zealand Joint Registry – Twenty-Two Year Report (2021).

#### Similarities

Structure or content of all reports shared similarities and differences. General speaking, all reports began with an Introduction which usually an overview of the registry history (update every time a new report publishes) and major adverse events which substantially affected the data collection and integrity e.g. COVID-19 pandemic. Statistics on two major joint replacements, hip and knee, followed. Details on the joint replacement were usually described following the sequence 1) overview, 2) primary replacement, and 3) revision.

#### Differences

Swedish registry mentioned 1) data quality completeness analysis, 2) patient-reported outcome measures, and 3) publications associated with the registry. National Joint Registry from the UK provided a summary of the important findings for the annual report, analysis of revisions in terms of implants, other kinds of joint replacements (ankle, elbow, shoulder), and a session on the effects of COVID-19 pandemic on the volume and waiting lists on joint replacement surgery. Australian Orthopaedic Association National Joint Replacement Registry provided a data snapshot on the major outcomes, data quality, summary of the impact of COVID-19 on joint replacement, patient reported outcome measures, and a session discussing the 10-, 15-, and 20-year prosthesis outcomes. New Zealand Joint Registry analysis additional arthroplasties—ankle, shoulder, elbow, lumbar disc replacement and cervical disc replacement. Patient reported outcome measures were discussed in the form of appendices.

#### Data collection for CUHK-PWH joint registry

Our joint registry has been proceeded and maintained by orthopaedic surgeons. Information from electronic prospective hospital records and clinical outcomes (health related quality of life questionnaires) were being entered during patient consultations. Surgical records were first recorded in information sheets as of busy surgical environments which refrained from real-time data entry. Surgical data were then entered into the electronic record system. Data were carefully extracted and entered into the registry by a member of the joint replacement surgical team, and further checked and maintained by a chief joint surgeon through a spreadsheet. Data confidentiality and safety has been well acknowledged by storing the database within departmental intranet with password protected.

#### Data collection from other registries

Major registries published their annual reports in their official websites and could obtain freely. Apart from the Australian registry which provided different supplementary files on separate topics e.g. lay summary, primary partial hip replacement supplementary report, primary partial knee replacement supplementary report, other reports provided all information in a single file.

#### Data synthesis and manipulation

Data from CUHK-PWH joint registry were native, as a result, data analysis could be performed on variables specific for our patients. Variables in common from the 4 registry annual reports were extract side by side. Examples of common variables were mean age, sex (% female and % male), American Society of Anesthesiologists (ASA) classification. Some data were best estimated from figures because the data were presented by charts only. For example, in Australian joint registry, percentages of cemented total knee replacement and percentage uncemented or hybrid knee replacements were best estimated from a chart because the results on the use of cement was described by a chart without a descriptive summary. Moreover, we tried to include as much as possible, therefore, we maximize the data integrity by including data even only available from 1 registry e.g. % ASA classification in male and female patients were only available from the UK registry report.

### Data analysis

Data from CUHK-PWH joint registry were described in terms of different descriptive statistics e.g. mean, standard deviation, minimum value, maximum value, range, median, quartiles. Numbers were presented in percentages because of the huge difference in the total numbers in registries. Data from other registries were presented as extracted, calculated (e.g. percentages of first revision after primary joint replacement from Australian registry were calculated by dividing the respective numbers by total number of total knee replacement (TKR) and unicompartmental knee replacement (UKR) cases), or chose the best alternatives close to the variables. Data were managed by Microsoft Excel spreadsheet and IBM SPSS 28.0 (Armonk, New York).

## Results

### Part 1: CUHK-PWH Joint registry

As of December 2020, CUHK-PWH registry captured a total of 2889 primary total knee replacements (TKR) and 879 were primary total hip replacements, of which 110 (3.81%) cases were revision TKR, and 107 (12.17%) cases were revision THR respectively. The reasons for the “unrecorded” or “missed” records were 1) patients received their revision procedures in local private sector 2) patients received their revision procedures overseas.

Mean body height and body weight were 157.7 cm and 62.8 kg respectively (Table 1). In a pooled analysis of body height and body mass index trajectories of children and adolescents from 1985 to 2019 in 200 countries and territories, the average body height was 167 cm and body weight was 67.8 kg [29]. Percentage distribution on BMI in knee and hip showed considerably different from a result of a government survey. The regional statistics on BMI distribution in Hong Kong was 8.6% with underweight, 68.5% Normal, 17.0% overweight and 3.7% were obese (2.2% were unknown/missing/outliers) [30]. Obese patients contributed the highest percentages in knee (70.0%) and hip (46.6%) patients, which was only 3.7% as of the above.

**Table 1:** Overview of CUHK-PWH registries until December 2020

	Knee	Hip
Body height (cm)		
Mean (SD) (Range)	156.1 (7.9) (135–179)	159.3 (9.9) (140–179)
Median (25^th^, 75^th^ percentile)	156 (151, 161)	159 (152, 168)
Body weight (kg)		
Mean (SD) (Range)	65.6 (11.9) (31–140)	60.0 (11.5) (33–103)
Median (25^th^, 75^th^ percentile)	64.9 (57, 73)	60 (52, 67)
BMI		
Mean (SD) (Range)	27.4 (4.4) (16.2–47.5)	24.7 (4.3) (15.8, 44.2)
Median (25^th^, 75^th^ percentile)	27.1 (24.3, 29.8)	24.7 (21.6, 27.3)
Underweight	1.6%	4.1%
Normal	11.7%	32.2%
Overweight	16.8%	17.1%
Obese	70.0%	46.6%
Steroid user	3.8%	4.4%
Smoker	7.5%	7.6%

Changes in implant selection in TKR and THR were tabulated in Table 2. To sum up, Our centre had only a handful of surgeons who dedicated to perform joint replacement surgery. Hence, we did not have a wide range of variability and techniques. Some changes did happen and were more subtle, such as changing from high-molecular-weight polyethylene to ultra-high-molecular-weight polyethylene and now high-density crosslinked polyethylene.

**Table 2:** Two major types of implants used in TKR and THR over the 35 Years in our centre

	Post-op > 20 years	Post-op 10–20 years	Post-op ≤ 10 years
Knee	PCA (61.9%)	Legacy (37.4%)	Legacy (50.8%)
	Others (23.8%)	IB II (28.6%)	PFC (15.9%)
Hip	Metal femoral head: 100.0%	Metal femoral head: 83.6%	Metal femoral head: 78.8%
	Ceramic femoral head: 0.0%	Ceramic femoral head: 16.4%	Ceramic femoral head: 21.2%

*TKR* Total Knee Replacement, *THR* Total Hip Replacement

We had little to no metal-on-metal bearing cases. Posterior-Stabilized (PS) TKR remained the main implant choice in our centre. We used Cruciate-Retaining (CR) TKR and Bi-cruciate Retaining (XR) TKR in recent years. The same applied for patella resurfacing. This was performed routinely until recent years. These recent changes had not been reflected in substantial numbers in our registry.

Tourniquet was used in all TKR cases. A drain was used during the early years and was omitted since the recent decade. The change in practice was largely due to the emerging clinical evidence on drain usage.

Surgery time of knee replacement was shorter than hip replacement (median surgical time = 110 vs. 125 min) (Table 3). On the other hand, the median drain output (ml) was higher in knee replacement surgery. Median number of days of hospital stay after hip replacement surgery was 2 days more than knee replacement surgery. There were 99.1% of patients went back own home or old age home after knee surgery, while as high as 73.8% stayed in convalescent hospitals in patients after hip replacement.

**Table 3:** Surgical details of CUHK-PWH registries until December 2020

	Knee (*N* = 879)	Hip (*N* = 2889)
Tourniquet time (minutes)		
Mean (SD) (Range)	89.5 (23.3) (7–175)	-
Median (25^th^, 75^th^ percentile)	86 (72, 105)	
Surgical time (minutes)		
Mean (SD) (Range)	113.1 (33.5) (12–1052)	131.1 (35.4) (20–300)
Median (25^th^, 75^th^ percentile)	110 (95, 130)	125 (107, 150)
Drain output (ml)		
Mean (SD) (Range)	682.1 (405.7) (0–4570)	487.6 (320.0) (10–1815)
Median (25^th^, 75^th^ percentile)	640 (370, 920)	440 (248, 670)
Transamin use		
Yes	93.1%	92.9%
Haemoglobin drop (g/dL)		
Mean (SD) (Range)	3.4 (1.4) (0–10)	3.9 (1.6) (0–24)
Median (25^th^, 75^th^ percentile)	3.3 (2.4, 4.3)	3.8 (2.9, 4.8)
Duration of hospital stay (days)		
Mean (SD) (Range)	10.4 (8.5) (2–207)	13.3 (12.6) (2–249)
Median (25^th^, 75^th^ percentile)	9 (7, 12)	11 (8, 15)
Discharge destination		
Back home	59.6%	26.2%
Old age home	39.5%	0%
Convalescent	0.9%	73.8%

**Fig. 1:**
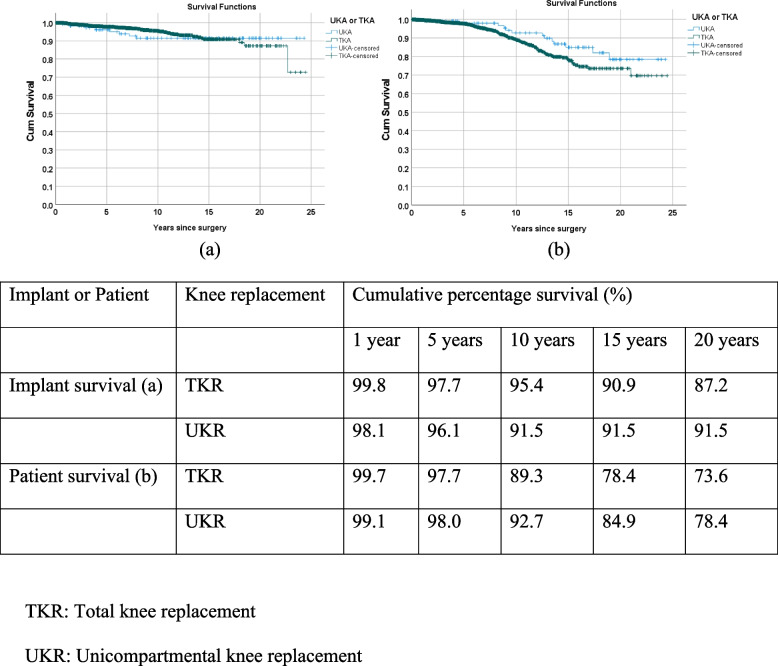
Kaplan Meier (KM) curves of cumulative percentage of primary knee implant survival at 1^st^, 5^th^, 10^th^, 15^th^, and 20^th^ year

**Fig. 2:**
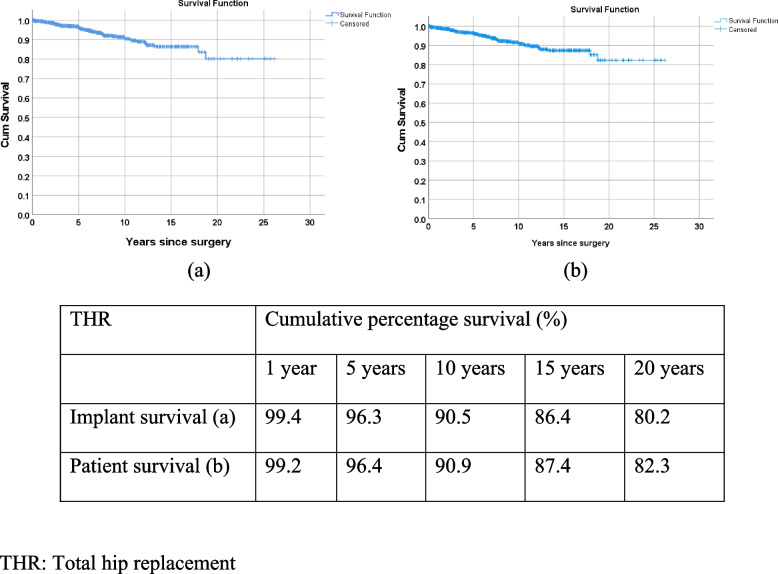
Kaplan Meier (KM) curves of cumulative percentage of primary hip implant and patient survival rates at 1^st^, 5^th^, 10^th^, 15^th^, and 20^th^ year

Survival analyses using Kaplan Meier product limit method were carried out on implant survivorship (from date of primary joint replacement surgery to date of first implant revision surgery) and patient survivorship (from date of primary joint replacement surgery to date of last seen (last seen status = Alive/Death)). In knee replacement surgery, cumulative implant survival percentages at the 10^th^ year was 95.4% (TKR) and 91.5% (UKR) and 87.2% (TKR) and 91.5% (UKR) at the 20^th^ year (Fig. 1). In hip replacement surgery, percentages of cumulative implant survival rate were 90.5% at the 10^th^ year and 80.2% at the 20^th^ year, and 90.9% and 82.3% at the 10^th^ and 20^th^ year for patient survivorship respectively (Fig. 2).

Clinical outcome scores were much improved (higher scores) at the last assessment follow-up compared with before surgery (Table 4).

**Table 4:** Clinical outcome scores before and after (latest) joint replacement in CUHK-PWH registry

	Knee		Hip	
	Pre-op	Latest	Pre-op	Latest
Knee Society Knee score	31.5 ± 13.8	90.8 ± 12.5	-	-
Knee Society Function score	46.4 ± 18.5	59.4 ± 27.2	-	-
Range of motion	91.6 ± 19.1	102.0 ± 16.2	-	-
Harris Hip Score	-	-	38.8 ± 13.1	80.2 ± 18.9

Latest: Data collected at the latest follow-up where data collection was possible

### Part 2: Comparing CUHK-PWH registry with other registries

The total numbers of records were shown in the first row in the overview of primary hip and knee replacement surgeries table (Table 5).

**Table 5:** Overview of primary hip and knee replacement surgeries at Year 2020

Joint	Knee					Hip				
Registry	CUHK-PWH	Sweden	UK	Australia	NZ	CUHK-PWH	Sweden	UK	Australia	NZ
Total number of records	2889	11,808	1.36 M^*^	0.91 M^#^	0.14 M^@^	879	12,049	1.25 M*	0.74 M^#^	0.16 M^@^
Age (Years)										
Mean (SD)	67.5 (8.0)	68.5 (9.10)	68.9 (9.6)	68.2 (9.5)	68.2 (8.7)	59.7 (13.2)	68.3 (10.2)	-	69.9 (12.6)	67.2 (11.35)
(Range)	(30 to 89)	(-)	(7 to 100)	(8 to 103)	(8 to 100)	(20 to 94)	(-)	(- to -)	(5 to 108)	(13 to 101)
Median (IQR)	68.0 (62–73)	- (-)	70 (63–76)	69 (-)	- (-)	61 (51–69)	- (-)	69 (61–76)	71 (-)	- (-)
Sex										
Male	73.0%	52.2%	43.7%	44.8%	48.5%	45.0%	43.5%	40.1%	43.8%	46.4%
Female	27.0%	47.8%	56.3%	55.2%	51.5%	55.0%	56.5%	59.9%	56.2%	53.6%
Joint replacement surgeries										
Knee										
TKR	97.5%	87.5%	87.9%	84.4%^a^	89.6%	25.4% (All cemented)	50.0% (All cemented)	31.3% (All cemented)	83.8%^2^ (All Total)	98.6% (All Total)
UKR	2.5%	12.5%	10.4%	12.9%^a#^	10.4%	50.7% (All uncemented)	32.5% All uncemented)	37.2% (All uncemented)	16.2%^2^ (All Partial)	1.4% (Resurfacing)
						11.0% (All hybrid)	8.0% (All hybrid)	22.7% (All hybrid)		0.1% (Hemiarthroplasty)
						12.9% (Others)	9.5% (Others)	8.8% (Others)		
TKR										
Cemented TKR	100.0%	91.0%	83.7%	66.7%	87.0%^b^				39.1% (Cemented)	5.0% (Cemented)
Uncemented TKR or Hybrid TKR	< 0.1%	9.0%	16.3%	33.4%	13.0%^b^				60.9% (Cementless)	50.0% (Cementless)
UKR										45.0% (Hybrid)
Cemented unicondylar	100.0%	40.4%	58.7%	-	58.8%					
Uncemented/hybrid unicondylar	< 0.1%	59.6%	41.3%	-	41.2%					

^*^ Cleaned data, primary knee and hip joint replacement procedures between 1 April 2003 and 31 December 2020 inclusive

^#^ Based procedures date up to and including 31 December 2020

^@^ Analyses data for the period between January 1999 and December 2020

^1^ Data for the period January 1999 to December 2020

^2^ Based on the sum of “All Primary Total” and “All Primary Partial”

IQR: Interquartile range

^a^ Calculated based on a total of 838,754 TKR and UKR cases up to and including 31 December 2020 in the Year 2021 Annual report

^b^ Best estimated data drawn from the figure “Comparison of proportions of cemented vs uncemented vs hybrid by year”

^a^^#^ UKR is a part of Primary Partial knee replacement

-: No data

#### Demographic characteristics

**Fig. 3:**
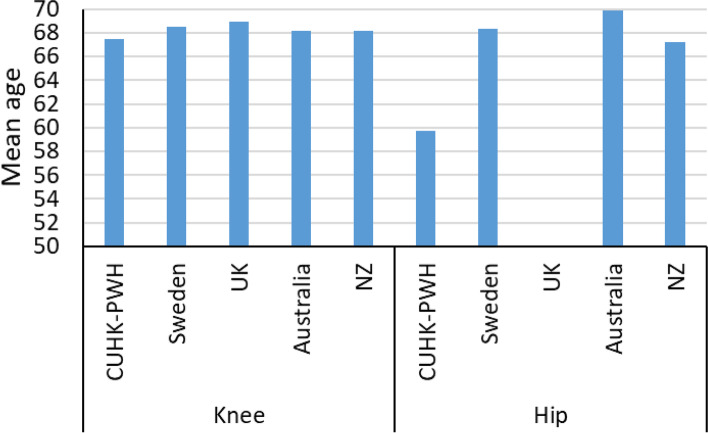
Mean age of the patients in the corresponding knee or hip registries

**Fig. 4:**
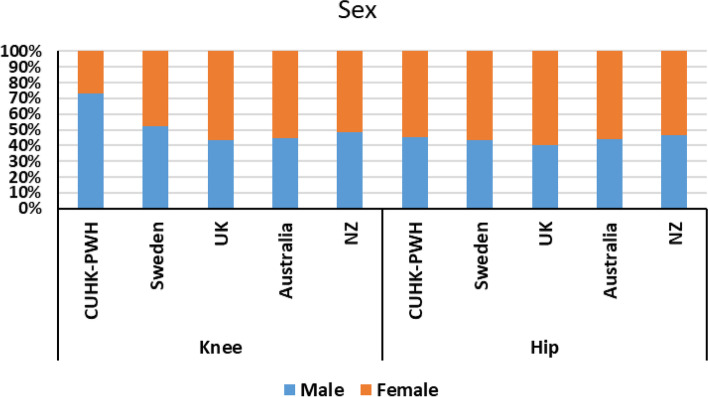
Sex of the patients in the corresponding knee or hip registries

**Fig. 5:**
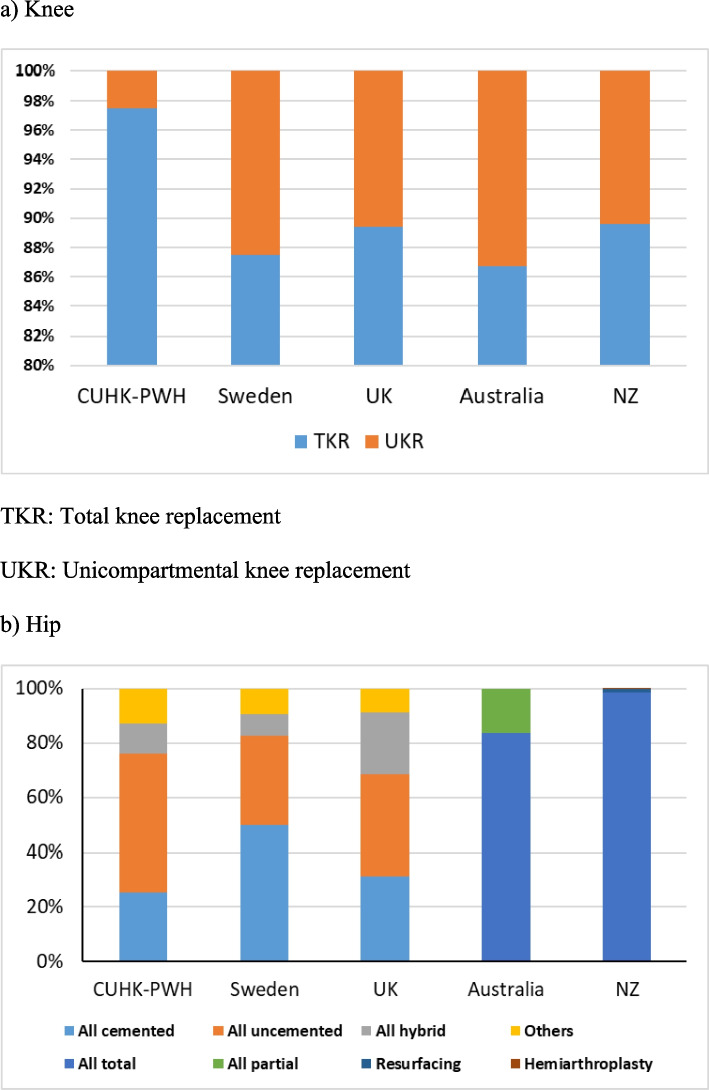
Joint replacement surgeries (%)

Mean ages of patients were the youngest age in CUHK-PWH for both knee (67.5 years old) and hip (59.7 years old) and oldest in UK (knee) and Australia (hip) (Table 5, Fig. 3). In knee, there were more female patients in the UK, Australia and NZ and more male patients in Sweden and CUHK-PWH (Fig. 4). Consistently more female received hip replacements. Percentages of TKR and UKR were similar among registries (Fig. 5). In TKR, uncemented (cementless) of hybrid were most popular in Australia (33.4%) and closed to none in CUHK-PWH (< 0.1%). Same observation in uncemented/hybrid UKR in CUHK-PWH which showed very few cases, while the percentages were similar in other registries (40.4% in Sweden to 41.3% in the UK). In hip, cemented and uncemented/hybrid/others were classified in 3 registries, and Sweden performed half-half between cemented surgery and other types.

#### American Society of Anesthesiologists (ASA) grade

Patients who underwent knee and hip replacement surgery showed the highest percentage with ASA grade 2, from 55.2% in Australia to 72.0% in the UK in knee and from 46.0% in Australia to 67.6% in UK (Appendix 4). Knee and hip patients with ASA grade 2 and 3 contributed very close to or higher than 90% in all registries (Sweden did not provide separate percentages in ASA grade 3 to 5 in knee).

#### Osteoarthritis as a reason and sole reason for primary joint replacement

Osteoarthritis contributed 95% of a reason to receive primary knee replacement in all registries, apart from CUHK-PWH with 91.4% (Appendix 5). UK registry provided another information on osteoarthritis being the sole reason for primary and this was a high as 96.6%. In hip, similar phenomenon has been found for osteoarthritis being the sole reason for primary surgery. Only 59.0% of records in CUHK-PWH showed the observation which is quite different from the others which were over 90%.

#### Revision after primary joint replacement, cumulative revision of implants, and reasons of revision

Percentage of CUHK-PWH patients requiring revision after primary knee replacement was 2.8%, which was similar to the statistics in the UK (3.0%) and Sweden (3.6%) (Table 6). Percentages were similar between Australia (8.7%) and NZ (7.9%). Percentages in hip were ranged from the lowest in the UK (3.0%) to NZ (13.9%).

**Table 6:** First revision after primary joint replacement

	Knee					Hip				
	CUHK-PWH	Sweden	UK	Aus^1^	NZ	CUHK-PWH	Sweden	UK	Aus^1^	NZ
Percentage of first revision after primary	2.8%	3.6%	3.0%	8.7%	7.9%	4.3%	9.4%	3.0%	7.7%	13.9%

^1^ Calculated based on a total of 838,754 TKR and UKR cases up to and including 31 December 2020 in the Year 2021 Annual report

Implant survivorship in terms of cumulative revision (%) and 95% confidence interval was provided by CUHK-PWH, UK and Australia. The 5-, 10-, 15- and 20-year % cumulative revision were basically similar among these 3 registries (Appendix 6). For example, % cumulative revision 10 years after knee replacement was 5.20% (CUHK-PWH), 4.13% (UK), and 4.80% (Australia), and % cumulative revision 10 years after hip replacement was 4.70% (CUHK-PWH), 4.28% (UK), and 4.40% (Australia).

Reasons for surgical revision had been summarized in Appendix 7. Aseptic loosening was found in 45.1% (knee) and 64.3% (hip) of revision cases in CUHK-PWH, 36.8% (TKR) and 14.1% (all kinds of hip revisions) in NZ, 38.2% (TKR) and 42.3% (all kinds of hip revisions) in the UK, 24.0% (TKR) and 48.0 (all kinds of hip revisions) in Sweden, and 14.0% (TKR) and 35.3% (all kinds of hip revisions) in Australia. Infection was found the lowest in the UK (TKR = 7.4%; Hip = 14.4%), followed by Australia (TKR = 17.9%; Hip = 17.5%) and NZ (TKR = 26.7%; Hip = 5.1%).

#### Mortality after primary replacement, 90-day mortality, and cumulative percentage survival after primary joint replacement

**Fig. 6:**
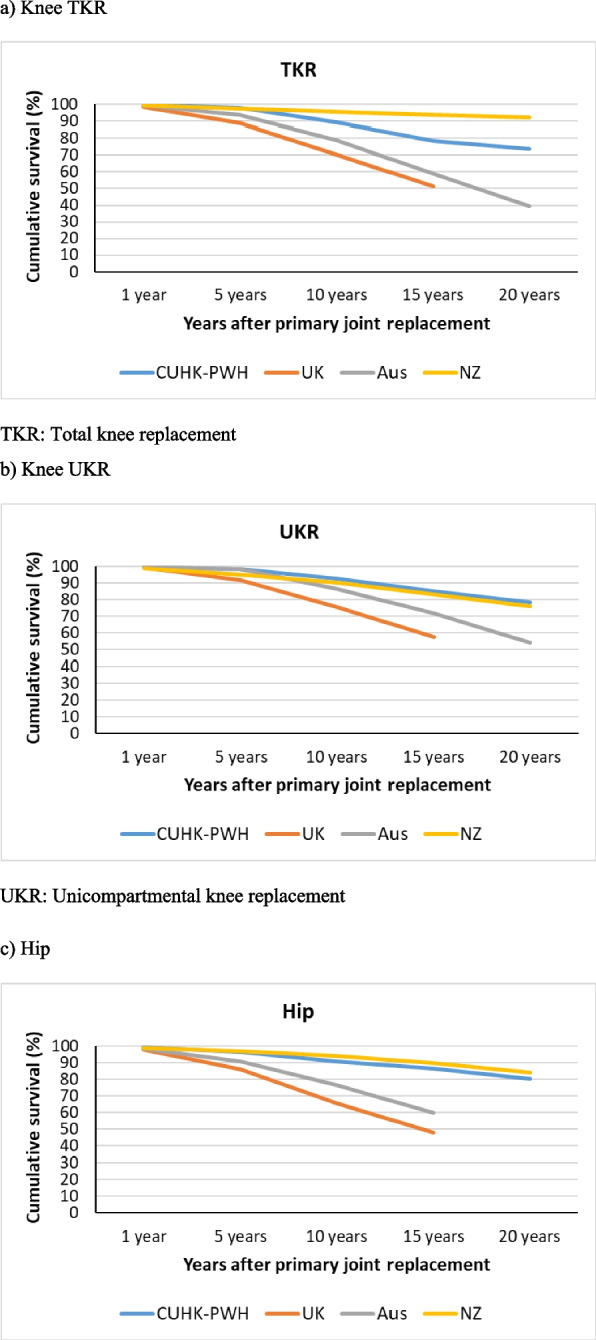
Cumulative percentage survival (%) corresponding to the number of years after primary joint replacement

Percentages mortality after primary joint replacement were available from CUHK-PWH, UK and Australia (Table 7). In TKR, the percentage was lowest in CUHK-PWH (13.9%) followed by UK (19.1%) and Australia (21.7%). In UKR, the percentages were similar between CUHK-PWH and UK, and higher in Australia. Mortality rate showed the highest percentage in Australia. Data on mortality within 90 days after primary joint replacement was available from the UK and Sweden (Appendix 8). UK data also provided information on mortality within 90 days after revision as well. Median age at revision for knee and hip was younger than primary replacement. Cumulative percentage survivorship after primary joint replacement was discussed (Table 8, Fig. 6). In TKR, percentages were similar in the first year after primary joint replacement among the 4 registries. UK was consistently lower than the other 3 registries, from the 5^th^ year to 15^th^ year (20^th^ year data not available from UK registry). Data from NZ reflected the best cumulative percentage survival with as high as 92.2% survivorship 20 years after primary TKR, 76.0% after primary UKR, and 84.2% after primary hip replacement.

**Table 7:** Mortality after primary joint replacement

	Knee					Hip					
	CUHK-PWH	Sweden	UK	Australia	NZ	CUHK-PWH	Sweden	UK	Australia	NZ	
Mortality after primary joint operation											
TKR	13.9	0.6%^4^	19.1%^1^	21.7%^2^	-^3^	5.9%	1.3%^4^	18.9%^1^	32.2%^5^	-^3^	Died
UKR	9.8		9.3%^1^	18.5%^2^	-^3^						

^1^ Based on the information provided from the UK National Joint Registry

^2^ Based on the data from “Mortality following Primary Knee Replacement by Class (Primary Diagnosis OA)”

^3^ Deceased data provided in the “The New Zealand Joint Registry Twenty Two Year Report (January 1999—December 2020)” without providing the necessary information

^4^ Mortality within 90 days of primary replacement

^5^ Based on “Number of Patients and Procedures Recorded by the Registry Between 1/9/1999 and 31/12/2020” in “Demographics of Hip, Knee and Shoulder Arthroplasty Supplementary Report” supplementary report

-: No data

**Table 8:** Kaplan Meier (KM) estimates of cumulative percentage survival (%) corresponding to the number of years after primary joint replacement

	1 year				5 years				10 years				15 years				20 years			
	CUHK-PWH	UK^1^	Aus^2^	NZ	CUHK-PWH	UK^1^	Aus^2^	NZ	CUHK-PWH	UK^1^	Aus^2^	NZ	CUHK-PWH	UK^1^	Aus^2^	NZ	CUHK-PWH	UK^1^	Aus^2^	NZ
Knee																				
TKR	99.7	98.6	99.3	99.2	97.7	89.1	93.5	97.3	89.3	70.2	79.1	95.7	78.4	51.2	59.2	93.8	73.6	-	39.5	92.2
UKR	99.1	99.2	99.6	98.6	98.0	91.8	98.0	94.8	92.7	75.6	86.4	90.2	84.9	57.3	71.8	83.0	78.4	-	54.1	76.0
Hip																				
All	99.4	97.6	98.4	98.7	96.3	85.7	90.8	97.0	90.5	65.7	76.6	94.1	86.4	48.1	60.0	89.8	80.2	-	-	84.2

^1^ Calculated using the data from “Mortality after primary knee surgery” and “Mortality after primary hip replacement surgery”

^2^ Based on the data from “Yearly cumulative percent survival of patients with primary knee replacement by class (Primary Diagnosis OA)”

## Discussion

With the emerging popularity of joint replacement surgery since the very first reported hip replacement considering to be one of the most successful orthopaedic interventions in Germany in 1891, more and more patients received knee and/or hip replacement surgeries which gained the necessity of developing an organized and well-structured database for data recording [31]. The importance and reasons of setting up a national joint registry has been discussed before. Over 35 years since the initiation of Swedish knee replacement registry, registries have been evolving in many different countries. After many years of development and data collection, the registry is an excellent source to carry out different kinds of data analysis. Major registries publish their annual reports in order to record patient information and provide data from surgical performance and longevity of replacement joint implants to evaluate performance outcomes of the surgeons who conduct the procedures. Synthesizing the annual reports give us an idea of what kinds of data should contain in a registry. There are many similarities and differences among different annual reports mostly because of geographical needs. That would be very useful to extract and synthesize similar information and compare the outcomes.

Information on demographic characteristics, surgical details, duration of hospital stay and discharge destination we share here aims at providing the possibilities to compare with other registries or studies. For sure there must be similarities and differences among registries/databases, for example, our mean and median BMI in knee and hip patients could be similar to patients within geographical regions and surrounding countries or countries with similar ancestor’s distribution, but at the same time that would be quite different from the other side of the world. Under the same principle, sharing the tourniquet time, surgical time and other surgical outcomes provide statistics for articles to reference to, while not judging one registry or study “outperforms” or “underperforms” the others. There are too many intrinsic and extrinsic factors in the respective administrative regions which give rise to the outputs. Above all, completeness of registry data is the most important factor to provide a true picture for clinicians and researchers to understand and improve surgical performance through data comparisons from different regions.

There are as high as 73.8% of our hip replacement patients stayed in convalescent hospitals. Our conventional practice was that many hip patients referred to convalescent hospitals for rehabilitation. This is different from the current practice in which the majority discharges to own home. The change in practice leading to the change in the percentages of discharge destination needs time to reflect in the joint registry. This, in turn, is limited by the data entry deadlog.

Patient-reported outcome measures (PROM) or patient-reported outcomes has been integrated into major registries. The introduction of PROM provides a very important source of patients’ feedback from whom requiring joint replacements which include administrative, research, clinical, and financial implications [32–35]. Our CUHK-PWH adapts Knee Society Knee score and Knee Society Function score for patients who underwent knee replacement surgery [36, 37], and Harris Hip score for patients who underwent hip replacement surgery [38]. Swedish registry initiates reporting PROM from 2002, and in 2019 results from EQ-5D [39], OMERACT-OARSI [40], VAS pain, EQ-VAS [39] and KOOS [41] are for knee and EQ-5D [39] and EQ-VAS [39] are for hip. The PROM described in National Joint Registry in the UK, was surprisingly Oxford Shoulder Score (OSS) (Dawson original version published in 1996 [42], revisited in 2009 [43]) (expected to report PROM related to knee and hip replacement surgeries). Australian registry began a pilot program collecting PROM data in 2017 and started rolling out the national PROM data collection practice to all surgeons and hospitals as they enrolled. PROM comprised EQ-5D [39] and EQ-VAS [39] for knee and hip, Oxford Hip Score [42], Oxford Knee Score [42], Oxford Shoulder Score [42], Hip disability and Osteoarthritis Outcome Score-12 (HOOS-12) [44], Knee injury and Osteoarthritis Outcome Score-12 (KOOS-12) [41], and other specific health-related questions. New Zealand registry described using “Oxford 12 Questionnaires” (a.k.a Oxford Knee [42], Hip [42] and Shoulder Score [42, 43]). Little is known on registries from Asian and middle east countries, either not visible in journals or not sharing the annual reports or documents on its kind online. The pilot project for setting up the Japan arthroplasty register was published without further elaborating the use of PROM [45]. This is hard to believe not to include any PROM in the Japan registry, however, no further information could be explored. It is obvious that, geographic location dictates the choice of PROM. The countries which the registries located are Europe countries or have a very close relationship with European countries (Australia and New Zealand). EQ-5D and Oxford series are the main choices of questionnaires. That also means that PROM results from registries can be directly compared although special caution should be made on how the questionnaires are delivered. A study using Mapping analysis (transfer to utility regression and response mapping) trying to develop mapping algorithms to estimate the EQ-5D from the Oxford Shoulder Score (OSS) owing to the fact that cost-utility analysis is calculated based on EQ-5D and that would be beneficial for registries collecting OSS without EQ-5D [46]. Our choice of using KKS and KFS for knee and HHS for hip is historical. Registries using EQ-5D and/or Oxford series as their major PROMs do not include KKS, KFS or HHS because they are somewhat complementary [47]. As a result, the choice of PROMs is somehow “splitting” the studies using either PROMs developed by European countries (EQ-5D and Oxford series) or America (KSS, KFS, HHS, WOMAC [48]). Developing and adapting one PROM which serve the purpose of both health-related quality of life measurements and cost utility analysis are highly recommended to “unify” the health-related quality of life research for patients who underwent knee and hip replacement surgery, making the results comparable among research groups in all parts of the world. We are not proposing a “replacement” to all well developed and adapted PROMs and propose to add this “universal PROM” along with the currently using PROMs. World Health Organization (WHO) should initiate this process for the sake of peoples’ health outcome improvement after joint replacement surgeries.

Maintenance of a national registry in terms of source of finding support and personnel involved dictates the success of a registry. It depends on the authorities involved. Majority of registries is maintained and supported by volunteering from chief and junior surgeons at pilot stage. Working out a decent, reliable, and up to date registry cannot solely depend on the scarce rest time within the harsh clinical environments. Our CUHK-PWH registry is maintained by adult joint replacement team without further funding injected by the government. Over 80% of Irish consultant orthopaedic surgeons and specialist registrars responded the source of funding on registry should be on government [49]. Major registries are funded by the government while maintain by orthopaedic associations and societies [50]. A registry is found to be successful when all patients can follow-up to their death, emigration or reoperation, as well as linking the joint registry with other registries like patient, cancer, prescription registers [51]. Obviously, financial support by the government means more administrative work on the registry governance and substantial outputs are necessary to follow to sustain the continuous support by the government. Issuing an annual report is an important output to reflect how we perform over the years (past), comparing this year’s performance with the past years (present) and forecast the statistics in the next destinated years, say 5 years (future). That would let all parties (government bodies, orthopaedics societies and clinicians) benefit from the registry and its external linkages.

Presence of registries from the Eastern side of the world (not including Australia and New Zealand) is still limited. In Korea, joint replacement related research uses the national data collected by the Health Insurance Review Agency (HIRA), although the number of articles using HIRA data is very few [52, 53]. Since the launching of the Japan Arthroplasty Register (JAR) for total hip arthroplasty (THA) in 2006 and setting up a pilot project reviewing the data between 2006 and 2011 [45], a report covering the annual data from year 2013 to 2016 revealing the current trend of THA in Japan was published in 2018 [54]. Thailand hip and knee joint registry ties with ASEAN arthroplasty joint registry [55] and we are waiting for a report on both registries. We expect the national joint registries from Asian countries are developing rapidly and will gain popularity soon.

### Limitations of this study

Missing data is inevitable in our registry. We try our best to maintain the registry in good shape amid huge amount of work has to be put. Some information from other registries has to be transformed which special attention on the use of these data should be paid. Data extraction is a very mind driven process and minor errors might be inevitable. Attention oughts to be paid on result interpretations because the total number of records in the 5 joint registries are hugely different from each other owing to the population sizes in these countries. We try to minimize the effect of population size by converting numbers to percentages where appropriate and possible. A unique advantage of our registry is that we apply the worldwide recognised implants in a different ethnicity population. Data stored in our registry, such as implant details, surgical procedures and outcomes of these implants can be used to compare with data from other registries. That would be very interesting to see the similarities and differences on various outcomes among ethnicity using the same sets of implants.

## Conclusion

The present study described the demographic characteristics, surgical details and patient related outcomes from our CUHK-PWH registry in 2020 and compared with the 2020 annual registry reports from Sweden, UK, Australia and New Zealand. Our results show that patient survivorship and outcome are comparable to data in international joint registries. This data set can update patients and clinicians on the revision rate for lower limb arthroplasty. The myth of revision surgery at 10 years is no longer accurate. Developing a world recognized and accepted patient reported outcome measure is recommended to facilitate PROM outcome comparisons. Funding from the government is recommended to support orthopaedic associations/societies to sustain the registry. Registries from Asian countries are expected to play a higher role in the future being at developing stage.

## Supplementary Information

**Additional file 1: Appendix 1.** Common variables (variables appeared in both Knee and Hip registry) collected in CUHK-PWH Registry.** Appendix 2.** Variables collected from Knee replacement patients.** Appendix 3.** Variables collected from Knee replacement patients.** Appendix 4.** Summary of ASA grading distribution from the 4 registries.** Appendix 5.** Percentages of patients presenting osteoarthritis as a reason or the sole reason for primary cause of joint replacement.** Appendix 6.** Kaplan Meier (KM) estimates of cumulative revision (95% confidence interval (CI)).** Appendix 7.** Reason for Knee and hip revision.** Appendix 8.** 90-day mortality after knee or hip revision.

## Data Availability

The datasets used and/or analysed during the current study are available from the corresponding author upon request.
