# Supplementary material for: Long-term survivorship and results in lower limb arthroplasty: a registry-based comparison study
Source: BMC Musculoskelet Disord. 2023 Apr 19;24:307. doi: 10.1186/s12891-023-06398-7 (PMC10113734; doi:10.1186/s12891-023-06398-7)
Supplement: Supplementary file 1 — Additional file 1: Appendix 1. Common variables (variables appeared in both Knee and Hip registry) collected in CUHK-PWH Registry. Appendix 2. Variables collected from Knee replacement patients. Appendix 3. Variables collected from Knee replacement patients. Appendix 4. Summary of ASA grading distribution from the 4 registries. Appendix 5. Percentages of patients presenting osteoarthritis as a reason or the sole reason for primary cause of joint replacement. Appendix 6. Kaplan Meier (KM) estimates of cumulative revision (95% confidence interval (CI)). Appendix 7. Reason for Knee and hip revision. Appendix 8. 90-day mortality after knee or hip revision. [file 12891_2023_6398_MOESM1_ESM.doc]

**Appendices**

Appendix 1. Common variables (variables appeared in both Knee and Hip registry) collected in CUHK-PWH registry

Personal identification number (HKID (Hong Kong Identity Card) number in our case), Full name (Surname and Forename), Sex, Age at data entry, date of birth, past medical history (PMH), body weight, body height, steroid use (Yes/No), smoker (Current/Ex-smoker/Never), Pre-op deformity (Nil/Varus/Valgus), Degree of Deformity, Pre-op Flexion, Diagnosis (entries like osteoarthritis, rheumatoid arthritis), surgeons involved (for internal use), Date of primary operation, side of operation, anaesthesia (e.g. GA), American Society of Anesthesiologists (ASA) Classification, Tourniquet Time (mins), operative time (mins), Drain output (ml), Transamin use (Yes/No), Transamin route & dose, Reason not for transamin, Pre-op Hb (g/dL), Hb drop (g/dL), Duration of post-op antibiotics (days), Complications (Yes/No), Duration of hospital stay, discharge destination (Home/Old age home)

Appendix 2. Variables collected from Knee replacement patients

Demographic variables: Previous knee operation

Surgical details: Cement, Model of Implant (Kinematic/PCA), Navigation used (Yes/No), Femur (Small/Medium), Tibial (Small/Medium), Patella (Small/Medium), Insert (mm), Distal Femur Cut (Degree + Valgus/Varus/Normal), Revision (Yes/No), Infection (Yes/No), Aseptic loosening (Yes/No)

Clinical outcomes (Patient reported outcome measures): Knee Society Knee Score (Pre-op and Latest), Knee Society Function Score (Pre-op and Latest)

Appendix 3. Variables collected from Knee replacement patients

Demographic variables: Previous Hip Operation

Surgical details: Gluteus Maximus Release (Yes/No), Iliopsoas Release (Yes/No), Anterior Capsulectomy (Yes/No), Previous Implant In-situ (Yes/No), Approach, Type of Shell (Omnifit PSL HA, Secur Fit HA PSL Cluster hole), Acetabular Cup Size (mm), Bearing Surface (Polyethylene, Ceramic), Acetabular Bone Graft (Nil/Morcelized), Acetabular Screw, Type of femoral stem (Secur-fit HA, Accolade, Accolade II), Femur Implant Size, Femur Bone Graft, Femoral Head Size, Type of Femoral Head (Metal/Ceramic), Capsule Repair (Modified Krackow's method/Nil)

Clinical outcomes (Patient reported outcome measures): Harris Hip score (Pre-op, follow-ups)

Appendix 4. Summary of ASA grading distribution from the 4 registries

| ASA | Male@ |  | Female@ |  | Total |  |  |  |  |
| --- | --- | --- | --- | --- | --- | --- | --- | --- | --- |
|  | CUHK-PWH | UK | HK | UK | CUHK-PWH | Sweden | UK | Australia | NZ |
| Knee |  |  |  |  |  |  |  |  |  |
| 1 | - | 13.2% | - | 10.1% | 11.6% | 19.9% | 11.4% | 6.3% | 11.0% |
| 2 | - | 70.4% | - | 73.2% | 69.6% | 65.6% | 72.0% | 55.2% | 63.5% |
| 3 | - | 16.0% | - | 16.4% | 18.5% | 14.5%  (3 to 5) | 16.2% | 37.5% | 25.0% |
| 4 | - | 0.4% | - | 0.3% | 0.3% |  | 0.3% | 1.1% | 0.5% |
| 5 | - | <0.1% | - | <0.1% | 0.0% |  | <0.1% | <0.1% | 0.0% |
|  |  |  |  |  |  |  |  |  |  |
| Hip |  |  |  |  |  |  |  |  |  |
| 1 | - | 18.0% | - | 14.0% | 19.7% | 22.6% | 15.6% | 7.9% | 16.0% |
| 2 | - | 65.0% | - | 69.4% | 62.9% | 61.8% | 67.6% | 46.0% | 59.0% |
| 3 | - | 16.4% | - | 16.2% | 16.6% | 15.4% | 16.2% | 40.2% | 24.0% |
| 4 | - | 0.6% | - | 0.5% | 0.8% | 0.2% | 0.6% | 5.8% | 1.0% |
| 5 | - | <0.1% | - | <0.1% | 0.0% | 0.0% | <0.1% | 0.1% | 0.0% |

ASA: American Society of Anesthesiologists

@ Data not available in Australia and NZ

-: No data

Appendix 5. Percentages of patients presenting osteoarthritis as a reason or the sole reason for primary cause of joint replacement

|  | Male |  |  |  | Female |  |  |  | Total |  |  |  |  |
| --- | --- | --- | --- | --- | --- | --- | --- | --- | --- | --- | --- | --- | --- |
|  | CUHK-PWH | UK | Australia | NZ | HK | UK | Australia | NZ | CUHK-PWH | Sweden | UK | Australia | NZ |
| Knee |  |  |  |  |  |  |  |  |  |  |  |  |  |
| Osteoarthritis as a reason for primary | - | 98.2% | - | - | - | 96.8% | - | - | 91.4% | >97.0% | 97.4% | 97.7% | 95.0% |
| Osteoarthritis as the sole reason for primary | - | 97.3% | - | - | - | 96.0% | - | - | - | - | 96.6% | - | - |
| Hip |  |  |  |  |  |  |  |  |  |  |  |  |  |
| Osteoarthritis as a reason for primary | - | 92.6% | - | - | - | 90.5% | - | - | 59.0% | 100.0%^*^ | 91.3% | 88.4% | 88.0% |
| Osteoarthritis as the sole reason for primary | - | 89.6% | - | - | - | 87.2% | - | - | - | - | 88.1% | - | - |

* Reported only total hip replacements performed due to osteoarthritis

Appendix 6. Kaplan Meier (KM) estimates of cumulative revision (95% confidence interval (CI))

|  | CUHK-PWH |  |  |  |  | UK^#^ |  |  |  | Australia |  |  |  |  |
| --- | --- | --- | --- | --- | --- | --- | --- | --- | --- | --- | --- | --- | --- | --- |
| Years after replacement | 1 | 5 | 10 | 15 | 20 | 1 | 5 | 10 | 15 | 1 | 5 | 10 | 15 | 20 |
| Knee |  |  |  |  |  |  |  |  |  |  |  |  |  |  |
| All | 0.01 (0.01-0.01) | 2.50 (2.43-2.58) | 5.20 (4.96-5.45) | 7.70 (6.50-8.90) | 8.30 (7.90, 8.60) | 0.49 (0.48-0.51) | 2.54 (2.51-2.57) | 4.13 (4.08-4.17) | 5.84 (5.75-5.93) | 1.00 (1.00, 1.00) | 3.30 (3.20, 3.30) | 4.80 (4.70, 4.90) | 6.50 (6.40, 6.60) | 8.10 (7.80, 8.50) |
| Hip |  |  |  |  |  |  |  |  |  |  |  |  |  |  |
| All | 0.01 (0.01-0.01) | 2.60 (2.54-2.66) | 4.70 (4.10-5.30) | 7.80 (7.10-8.40) | 9.10 (8.50-9.70) | 0.81 (0.79-0.83) | 2.16 (2.13-2.19) | 4.28 (4.23-4.33) | 6.89 (6.78-6.99) | 1.60 (1.50, 1.60)^1^ | 2.90 (2.80, 3.00)^1^ | 4.40 (4.30, 4.50)^1^ | 6.50 (6.30, 6.70)^1^ | 9.00 (8.30, 9.80)^1^ |

* Data not provided in NZ 2012 report

# UK registry report did not provide information at the 20^th^ year

^1^ Based on “Revision of Primary Total Conventional Hip Replacement (Primary Diagnosis OA)” being the majority in hip replacement surgery

Appendix 7. Reason for Knee and hip revision

|  | CUHK-PWH | Sweden |  | UK | Australia |  | NZ |  |
| --- | --- | --- | --- | --- | --- | --- | --- | --- |
| Knee | TKR | TKR | UKR | TKR | TKR | UKR | TKR | UKR |
| Aseptic loosening | 45.1% | 24.0% | 25.0% | 38.2% | 14.0% | 33.4% | 36.8% | 26.7% |
| Instability | <0.1% | 16.5% | 13.0% | 17.4% | 33.6% | 1.5% | 0.1% | - |
| Pain | <0.1% | Not categorized | Not categorized | 14.9% | 0.9% | 7.8% | 28.7% | - |
| Implant wear | 9.0% | 3.5% | 8.0% | 14.0% | Not categorized | 1.5% | <0.01% | - |
| Other indication | Not categorized | 20.0%^1^ | 12.0%^2^ | 11.2% | Not categorized | Not categorized | Not categorized | - |
| Malalignment | 0.0% | Not categorized | Not categorized | 7.6% | 0.7% | 1.3% | Not categorized | - |
| Infection | 33.8% | 30.0% | 3.0% | 7.4% | 17.9% | 4.8% | 26.7% | - |
| Periprosthetic fracture | <0.1% | 4.0% | 3.5% | 4.5% | Not categorized | 2.8% | 3.1% | - |
| Dislocation/Subluxation | 3.0% | Not categorized | Not categorized | 4.1% | Not categorized | 3.3% | Not categorized | - |
| Stiffness | 0.0% | Not categorized | Not categorized | 5.7% | Not categorized | Not categorized | <0.01% | - |
| Progressive arthritis | <0.1% | 0.1% | 35.5% | 14.9% | Not categorized | 36.1% | Not categorized | - |
|  |  |  |  |  |  |  |  |  |
| Hip |  | All revisions |  |  | All revisions |  | All revisions |  |
| Aseptic loosening | 64.3% | 48.0% | - | 42.3% | 35.3% | - | 14.1% | - |
| Instability | 0.0% | 13.0% | - | Not categorized | 0.7% | - | Not categorized | - |
| Pain | 0.4% | Not categorized | - | 15.4% | 2.0% | - | 5.3% | - |
| Implant wear | 5.1% | Not categorized | - | 12.5% | 2.1% | - | Not categorized | - |
| Other indication | Not categorized | 6.0% | - | 6.8% | 0.9% | - | Not categorized | - |
| Malalignment | 0.7% | Not categorized | - | 4.8% | 0.6% | - | Not categorized | - |
| Infection | 5.5% | 21.0% | - | 14.4% | 17.5% | - | 5.1% | - |
| Periprosthetic fracture | 1.6% | 12.0% | - | 10.9% | 12.4% | - | 4.7% | - |
| Dislocation/Subluxation | <0.1% | Not categorized | - | 14.7% | 14.5% | - | 7.8% | - |
| Stiffness | 0.0% | Not categorized | - | Not categorized | Not categorized | - | Not categorized | - |
| Progressive arthritis | <0.1% | Not categorized | - | Not categorized | Not categorized | - | Not categorized | - |

^1^ Patella: 12.0%, Others: 8.0%

^2^ Patella: 1.5%, Others: 10.5%

Appendix 8. 90-day mortality after knee or hip revision

|  | Cumulative percentage mortality at 90 days | Median age (IQR) | % Male | Cumulative percentage mortality at 90 days | Median age (IQR) | % Male |
| --- | --- | --- | --- | --- | --- | --- |
| UK | Knee |  |  | Hip |  |  |
| Primary replacement | 1.00 (0.90-1.12) | 73 (65-79) | 46.9 | 1.90 (1.80-2.00) | 74 (66-80) | 44.2 |
| Revision | 0.72 (0.65-0.81) | 68 (61-75) | 45.1 | 1.42 (1.30-1.54) | 69 (61-77) | 42.5 |
|  |  |  |  |  |  |  |
| Sweden |  |  |  |  |  |  |
| Primary replacement | 0.64^1^ | - | - | 1.30^2^ | - | - |

^1^90-days mortality after primary total knee replacement due to osteoarthritis; weighted %; osteoarthritis per region 2017–2019

^2^90-days mortality after primary hip replacements due to osteoarthritis; weighted %; osteoarthritis per region 2017–2019
